# DOG-1 as a Myoepithelial Marker in Discriminating Invasive Breast Carcinomas From Non-Invasive Breast Lesions

**DOI:** 10.7759/cureus.39676

**Published:** 2023-05-29

**Authors:** Sushmitha Perumandal, Divya Madhuri Ponnaboina, Mohmed Chand Moula, Yadavalli RD Rajan

**Affiliations:** 1 Pathology, Osmania Medical College, Hyderabad, IND; 2 General Surgery, Bhaskar Medical College, Hyderabad, IND

**Keywords:** p63 oncogene, invasive breast carcinoma, myoepithelial marker, benign breast disease, dog-1

## Abstract

Introduction

The most common cancer in women is, by far, breast cancer. The incidence and mortality of breast cancer must be reduced by a multidisciplinary strategy that includes education campaigns, preventive measures, screening programmes for early diagnosis, and the availability of treatment facilities. The use of immunohistochemical (IHC) stains with relative specificity for myoepithelial markers has become a mainstay of standard diagnostic breast pathology because the presence and distribution of myoepithelial cells might differ greatly amongst the distinct breast proliferation. Although it has also been reported that DOG1 is expressed in other mesenchymal tumours, DOG1 has been demonstrated to be sensitive and specific for the detection of gastrointestinal stromal tumors (GISTs). Both myoepithelial cells (MECs) and luminal epithelial cells have occasionally displayed DOG1 immunoreactivity in the breast.

Materials and methods

This prospective cross-sectional study was done in the Department of Pathology at Osmania General Hospital, Hyderabad on 60 cases from June 2017 to June 2019. Female patients with different breast lesions including benign proliferating lesions, ductal carcinoma in-situ (DCIS), and invasive carcinoma breast cases were included in the study. Inflammatory lesions, mesenchymal, and metastatic tumors were excluded. IHC expression of DOG1 as a myoepithelial marker to discriminate invasive from non-invasive breast lesions was evaluated and correlated with clinicopathological features.

Results

The mean age of the study population was 33.67 ± 8.48 in the benign group and 54.43 ± 12.84 in the malignant group. Fifty percent (15) of the patients with benign lesions belonged to the age group 20-30 years, whereas 26.7% (8) of the patients with malignant lesions belonged to the age group 61-70 years. DOG-1 expression was strongly positive in fibroadenoma, ductal hyperplasia, fibrocystic disease, whereas strongly negative in malignant disease of the breast (*p* < 0.0001). P63 expression was strongly positive in benign breast diseases and strongly negative in malignant diseases (*p *< 0.0001).

Conclusion

DOG1 seems to be similar to p63 as a myoepithelial cell marker both in normal breast tissue and in benign lesions. DOG1 is strongly positive in benign breast diseases and strongly negative in malignant breast diseases. Hence, it can be considered as a useful myoepithelial marker in differentiating invasive breast carcinoma and non-invasive breast lesions.

## Introduction

Breast cancer is by far the most frequent cancer among women with an estimated 2 million new cancer cases diagnosed in 2018 (23% of all cancers), and ranks second overall (10.9% of all cancers) [1]. Breast cancer has ranked number one cancer among Indian females with age adjusted rate as high as 25.8 per 100,000 women and mortality 12.7 per 100,000 women [2]. The vast majority of the lesions that occur in the breast are benign. The term “benign breast disease” encompasses a heterogeneous group of lesions that may present a wide range of symptoms or may be detected as incidental microscopic findings [3]. At the light microscopic level, two distinct cell populations can be recognized lining the human mammary ductal and terminal duct-lobular units namely, a luminally located layer of polarized epithelial cells and a basally located layer of myoepithelial cells. These cell populations display significant differences in normal function and protein expression profiles. Because the presence and distribution of myoepithelial cells can significantly differ between the various breast lesions, the use of immunohistochemical (IHC) stains with relative specificity for myoepithelial markers has become a staple of routine diagnostic breast pathology [4]. DOG1, also known as TMEM16A, FLJ10261, ORAOV2, and anoctamin, was identified as a typical finding on gastrointestinal stromal tumors (GISTs). DOG1 is a sensitive and specific marker for detecting GISTs, although expression of DOG1 in other mesenchymal tumors has also been reported. In the breast, both myoepithelial cells (MECs) and luminal epithelial cells have shown DOG1 immunoreactivity in a few cases [5]. This study aims to study the IHC expression of DOG1 as a myoepithelial marker to discriminate invasive from non-invasive breast lesions and to correlate the IHC expression of DOG1 with clinicopathological features.

## Materials and methods

This prospective cross-sectional study was done in the Department of Pathology at Osmania General Hospital, Hyderabad on 60 cases from June 2017 to June 2019. Female patients with different breast lesions including benign proliferating lesions, ductal carcinoma in-situ (DCIS) and invasive carcinoma breast cases were included in the study. Inflammatory lesions, mesenchymal and metastatic tumors were excluded from the study. The specimens were fixed in 10% neutral buffered formalin. For the sake of convenience, non-invasive breast carcinomas (DCIS) were included in the benign/non-invasive category. They were examined grossly according to the standard guidelines, with special emphasis on the lesion's tumor size and lymph node status. The specimens were grossed and sections were taken from representative sites. These sections were then processed in a tissue processor and embedded in paraffin wax. Four-to-five-micron thickness sections were prepared from the corresponding paraffin blocks, one on the albumin-coated slide for hematoxylin and eosin (H&E) staining and the other on poly-L-lysine coated slide for immunohistochemical staining. Immunohistochemical staining was done using the peroxidase-anti-peroxidase method according to the protocol described by BioGenex (Fremont, CA, USA). The following antibody clones were used: DOG-1 (Mouse Monoclonal antibody-PM127 in PBS carrier protein and preservative) and P63 (Mouse monoclonal antibody 4A4 in PBS carrier protein and preservative).

DOG-1 is a cytoplasmic and membranous stain and p63 is a nuclear stain. Immunoreactivity for each antibody was assessed separately. Immunoreactivity for each antibody was assessed separately and the intensity of DOG1 and p63 was classified as follows: weak or no staining received a score of 1, moderate staining received a score of 2, and strong staining received a score of 3 [5]. Data were entered into Excel sheets and the data were analyzed using Microsoft Excel 2013 (Microsoft® Corp., Redmond, WA, USA). Categorical data were represented in the form of frequencies and continuous data were represented as mean and standard deviation. A *p*-value of < 0.05 was considered statistically significant. The study abides by the guidelines laid by the Declaration of Helsinki. Ethical committee clearance was obtained from the Institutional Ethics Committee, Osmania Medical College (ECR/300/Inst/AP/2013/RR-16).

## Results

The mean age of the study population was 33.67 ± 8.48 in the benign and non-invasive breast lesions group and 54.43 ± 12.84 in the invasive lesions group. Fifty percent (15) of the patients with benign lesions belonged to the age group 20-30 years, whereas 26.7% (8) of the patients with malignant lesions belonged to the age group 61-70 years. Twenty-five patients had breast lump as the presenting symptom in the benign lesion and non-invasive breast lesions group and 22 patients had breast lump as the presenting symptom in the invasive lesions group. In the benign and non-invasive lesions group, 46.70% (14) had lesions in the left side of the breast and 43.30% (13) had lesions in the right side of the breast and the rest were bilateral (3). In the invasive group, 63.30% (19) had lesions in the right side of the breast and the rest were left-sided lesions (11).

Histopathological diagnosis of benign breast diseases and non-invasive breast lesions is shown in Table 1.

**Table 1:** Histopathological diagnosis of the benign and non-invasive breast disease cases. DCIS: Ductal carcinoma in-situ

Diagnosis	Number of cases
Ductal hyperplasia	3
DCIS	3
Fibrocystic disease	4
Phyllodes	4
Adenosis	2
Fibroadenoma	14

A comparison of histopathological diagnosis with the intensity of DOG1 expression is shown in Table 2.

**Table 2:** Comparison of histopathological diagnosis with the intensity of DOG1 expression DCIS: Ductal carcinoma in-situ

Nature of disease	Histopathological diagnosis	DOG-1 Expression Intensity			Total
		Negative	Weak Positive	Strong Positive	
Benign and non-invasive disease	Fibroadenoma	0	3	11	14
	Adenosis	0	1	1	2
	Phyllodes	2	0	2	4
	Fibrocystic disease	1	1	2	4
	DCIS	1	0	2	3
	Ductal hyperplasia	0	1	2	3
Invasive disease	Invasive Carcinoma	28	2	0	30
Total		32	8	20	60
Fischer exact = 55.61, p < 0.0001					

A comparison of histopathological diagnosis with the intensity of P63 expression is shown in Table 3.

**Table 3:** Comparison of histopathological diagnosis with the intensity of P63 expression DCIS: Ductal carcinoma in-situ

Nature of disease	Histopathological diagnosis	P63 Expression Intensity			Total
		Negative	Weak Positive	Strong Positive	
Benign and non-invasive disease	Fibroadenoma	0	3	11	14
	Adenosis	0	0	2	2
	Phyllodes	1	1	2	4
	Fibrocystic disease	0	1	3	4
	DCIS	1	0	2	3
	Ductal hyperplasia	0	0	3	3
Invasive disease	Invasive Carcinoma	30	0	0	30
Total		32	5	23	60
Fischer exact = 66.78, p < 0.0001					

**Figure 1:**
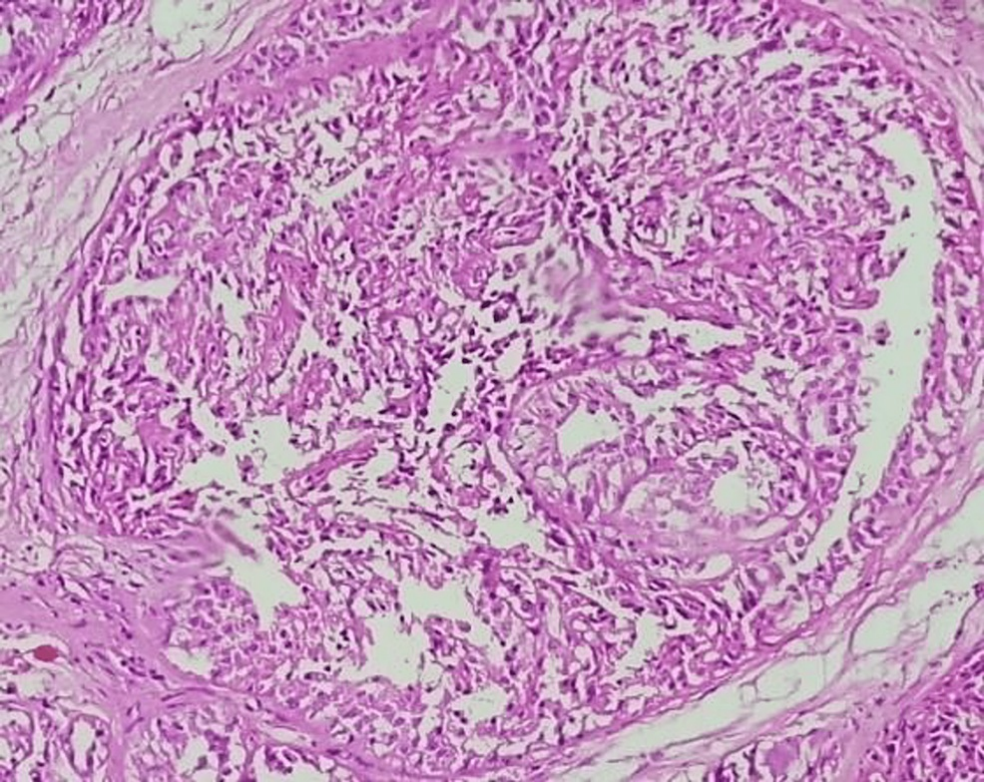
Hematoxylin & Eosin (H&E) Stained, 10x zoom image showing Ductal Carcinoma In-situ: proliferation of epithelial cells with low nuclear grade in solid pattern admixed with nuclear debris.

**Figure 2:**
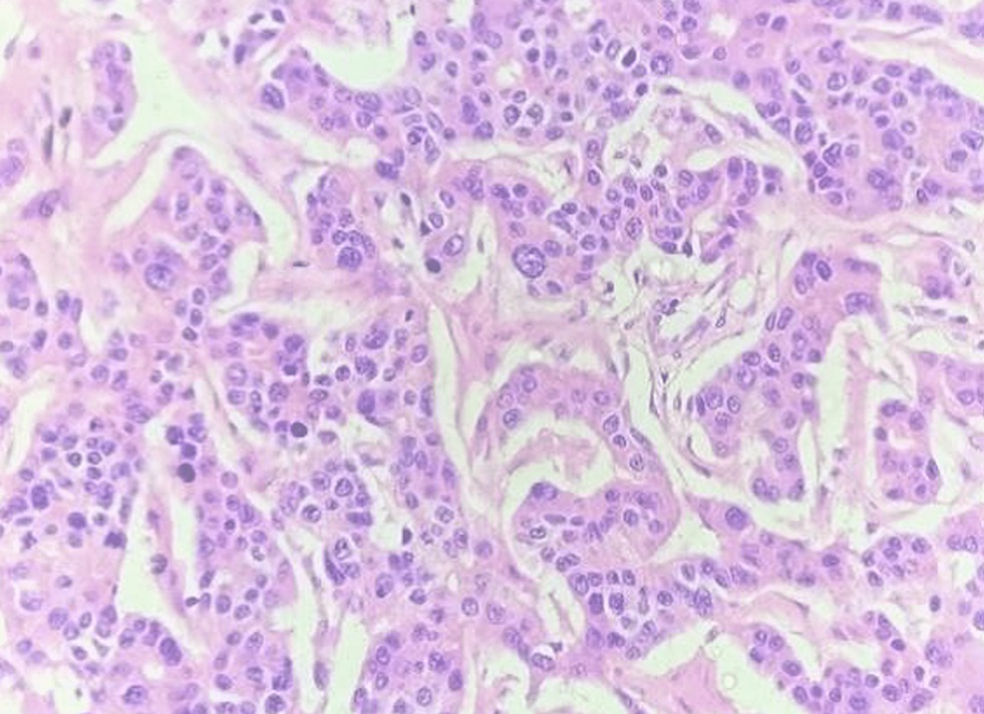
H&E, 10x image of Invasive Breast Carcinoma: Cords and trabeculae of tumor cells with intervening scant stroma. H&E: Hematoxylin and Eosin

**Figure 3:**
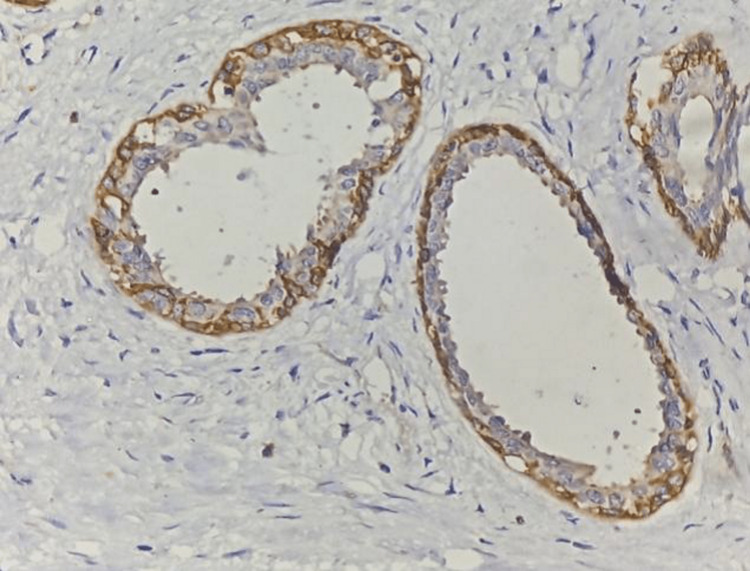
40x image of fibroadenoma showing DOG1 strong positive (Membranous) in myoepithelial cells.

**Figure 4:**
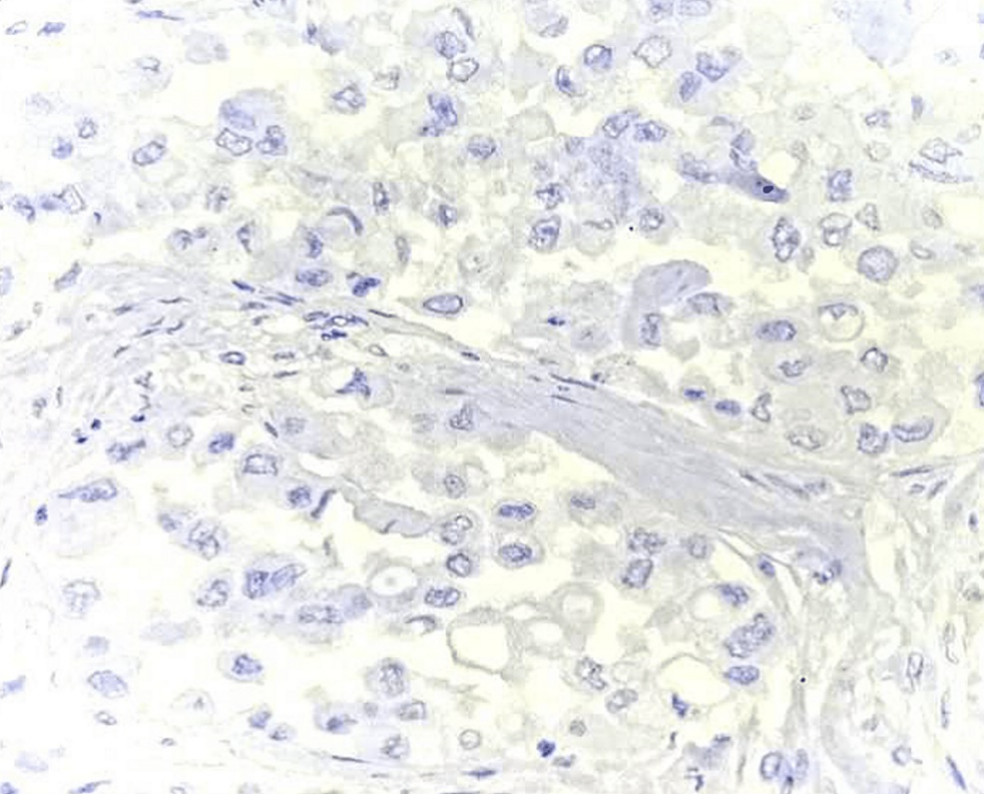
Histopathological examination (HPE) (40x image) showing Invasive Carcinoma of the breast NST, DOG1 Negative NST: no special type

Histopathological images of the slides are shown in Figures 1-4 below.

## Discussion

This prospective study was conducted on 60 female patients in the upgraded Department of Pathology, at Osmania Medical College, Hyderabad. Out of 60 female patients with breast lesions, 30 presented with benign and non-invasive lesions and 30 with invasive lesions. In the present study, the mean age of patients with benign non-invasive lesions was 33.67 ± 8.48 years and that of patients with invasive lesions was 54.43 ± 12.84 years which indicates carcinomas were seen in elderly patients of age above 50. In the present study, age groups of benign and non-invasive breast disease patients were categorized in which 50% (15) of patients belonged to the age group of 20-30 years, 26.7% (8) to the age group 31-40 years, and 23.3% (7) belonged to the age group of 41-50 years, which indicates that benign lesions are more common in younger age group. Similarly, in a study done by Neeli et al. in 185 patients, the mean age of all patients with benign and non-invasive lesions was 34 years and 45.83% (33) belonged to the age group 21-30 years which is nearer to our study age group and 25% (18) belonged to 11-20 years age group, 18.05% (13) were among 31-40 years age group and 11.12% (8) belonged to 41-50 year age group [6]. Verma et al. conducted a study on 151 breast cases on “The Role of p63 expression in non-proliferative and proliferative lesions of the breast” which showed a mean age of 31.3 years [7].

In our study, age groups of patients with invasive breast lesions were categorized in which 26.7% (8) belonged to the age group 61-70 years which was the elderly age group, 23.3% (7) belonged to the age group 41-50 years, and 51-60 years age group respectively, 10% (3) to age group 71-80 years and 31-40 years respectively and 6.7% (2) i.e., very few belonged to age group 20-30 years and these results correlated well with the study by Neeli et al. [6]. In the present study, among the benign and non-invasive breast diseases, 25 (83.33%) cases presented with a breast lump, and five (16.67%) cases presented with a breast lump and pain. These results correlated well with the study by Ramesh and Bookya in which there were 250 benign breast cases, of which 170 (68%) presented with a breast lump, 60 (24%) presented with a breast lump and pain, 15 (6%) presented with a breast lump, pain and nipple discharge and 5 (2%) presented with nipple discharge [8]. Among the invasive breast lesions, in the present study, 22 (73.33%) out of 30 cases presented with breast lump only, five (16.67%) presented with a breast lump and pain, and three (10%) presented with a breast lump and nipple discharge. These results correlated well with Rathi et al. study in which 21 cases were malignant. Out of 21 cases, 17 (80.96%) cases presented with breast lump alone, two (9.52%) cases presented with nipple discharge alone, one (4.76%) presented with lump and pain and one (4.76%) presented with lump, pain and nipple discharge [9]. In the present study, the laterality of the breast involved in benign lesions includes 46.70% (14) with left-side involvement, 43.30% (13) with right-side involvement of the breast, and 10% (3) of them with bilateral involvement of breast. Sukanya et al. have done a study on 60 benign breast diseases which showed that 46.67% (28) cases had left-side involvement which was similar to the present study [10]. In the present study, clinical diagnosis of 30 benign breast lesions included 76.70% (23) of fibroadenomas, 13.30% (4) of fibrocystic disease and 10% (3) of phyllodes cases which correlated well with Ramesh and Bookya's study [8]. Clinical diagnosis of invasive breast disease in the present study showed that 93.30% (28) belonged to invasive carcinoma breast and 6.70% (2) belonged to fibrocystic disease.

Histopathological diagnosis

In our study, among the benign and non-invasive breast diseases, 14 out of 30 were diagnosed as fibroadenoma, four cases were diagnosed as fibrocystic disease and phyllodes respectively, three cases were diagnosed as ductal hyperplasia and DCIS respectively and two cases were diagnosed as adenosis. Neeli et al. [6] have done a study on benign breast disease in which 11 cases were diagnosed as an inflammatory disease, 70 were diagnosed as fibroadenoma, 23 as fibrocystic disease, 36 as fibroadenosis, nine cases as gynecomastia and one case each diagnosed as phyllodes and duct papilloma, respectively. In a study done by Shashikala et al., 37 cases were diagnosed as fibroadenoma, 23 cases as fibroadenosis, four cases as phyllodes, seven cases as breast abscess, two cases as duct ectasia, three cases as lipoma, 14 cases were diagnosed as fibroadenoma with fibrocystic change and one case with accessory breast [11]. Clinicopathological correlation of the benign and non-invasive breast disease in the present study shows that 13 out of 23 cases of fibroadenoma (clinically diagnosed) were diagnosed as fibroadenoma histopathologically, three out of 23 diagnosed as ductal hyperplasia, two were diagnosed as adenosis, two as phyllodes, two as fibrocystic disease and one case as DCIS, where the accuracy of clinical diagnosis was 56.57%. Two out of four cases of fibrocystic disease (clinically diagnosed) were diagnosed histopathologically as fibrocystic disease with a clinical accuracy of 50% and two were diagnosed as DCIS, two out of three cases were diagnosed as phyllodes with an accuracy of 66.66% and one case out of three diagnosed as fibroadenoma which was statistically significant (p-value = 0.017). In a study by Ramesh and Bookya, out of 186 cases diagnosed clinically as fibroadenoma, 142 cases were finally diagnosed as fibroadenoma which shows clinical accuracy of 76.34%, 17 cases were diagnosed as proliferative breast disease with florid hyperplasia, 13 cases were diagnosed as proliferative breast disease with atypia, five cases were diagnosed as intraductal papilloma, four cases were diagnosed as duct ectasia, two cases as phyllodes and one case as simple cyst [8]. In the present study, TNM (Tumor, Node, Metastasis) staging in 30 breast carcinoma cases showed 46.50% (14) in stage T2 N0 M0, 16.70% (5) in stage T2 N1 M0 and T3 N0 M0 respectively, 6.70% (2) cases each in stage T2 N2 M0, T3 N1 M0 and T3 N2 M0 respectively. American Joint Committee on Cancer (AJCC) staging of these patients showed 46.70% (14) were in stage 2A, 33.30% (10) were in stage 2B and 20% (60) were in stage 3A. These results correlated well with Chuang et al. study in which 33% (5206) were in AJCC stage 1, 47% (7477) were in stage 2 and 20% (3198) were in stage 3 [12].

In the present study, DOG-1 expression in invasive and non-invasive breast diseases was compared with p63 expression. Out of 14 cases of fibroadenoma, 11 were strong positive for DOG1 and three were weak positive. Similarly, 11 were strong positive and three were weak positive for p63 expression which showed no significant difference between DOG-1 and p63. Two cases of adenosis also showed positivity for DOG1 expression in which one case was strong positive and the other one was weak positive. But p63 showed strong positivity in both these cases. Among four cases of phyllodes, two were completely negative for DOG1 expression and two were strong positive for DOG1 expression. p63 was strong positive in two cases, one case was weak positive and another one was completely negative for p63 expression. Compared to p63, DOG1 was completely negative only in one case. Out of four cases of fibrocystic disease, two cases were strong positive, one weak positive and one negative for DOG1 expression. p63 was strong positive in three cases and one case was weak positive. Compared to p63, DOG-1 was negative only in one case. Out of three cases of DCIS, two were strong positive and one case was negative for both DOG1 and p63. There were three cases of ductal hyperplasia of which two cases showed strong positivity and one case showed weak positivity for DOG1 expression but p63 was strongly positive in all the three cases.

Finally, out of 30 benign cases, 26 cases showed positivity for DOG1 expression which is 86.66% and 28 cases showed positivity for p63 expression with a specificity of 93.33%. Out of 30 cases of invasive carcinomas of the breast, 28 cases were negative but only two cases showed weak positivity, i.e, DOG1 expression was positive in only 6.66% of cases of invasive breast disease which indicates that DOG1 expression in non-invasive lesions is statistically highly significant (p < 0.001). All 30 cases of invasive carcinomas were completely negative for p63 with a 0% specificity which is statistically highly significant (p<0.001). Compared to p63, DOG1 was weak positive only in two cases. In previous research, Cheng et al. conducted a study on DOG1 expression in 160 cases of different breast lesions. Seventeen out of 20 adenosis cases were strong positive and the remaining three were weak positive. Out of 20 cases of intraductal papilloma, 19 cases were strong positive and one case was weak positive, whereas 20 cases of intraductal papilloma showed strong positivity for p63 with a specificity for benign lesions as 97.50% which is higher than our study [5]. All the 60 cases of invasive carcinoma breast were negative for DOG-1 which indicates that DOG1 expression is seen only in benign lesions. Fifty-nine out of 60 invasive carcinoma cases showed negativity and one case shows weak positive for p63 expression with a specificity of 1.66% which is higher than our study [5]. Goto has studied the role of DOG1 immunohistochemistry in dermatopathology which also showed that among 21 cases of fibroadenoma breast, nine cases were positive for DOG1 expression with specificity of 43% [13]. In a study conducted by Swalchick et al., nine out of 11 cases of fibroadenoma show DOG1 positivity with a specificity of 82% [14]. Loss of the outer MEC layer is the hallmark of invasive breast carcinoma. Therefore, the identification of MECs is important for the differential diagnosis of breast lesions, and IHC is an effective method for identifying these cells [15]. Clinically, the most commonly used immunohistochemical markers are cytoplasmic antigens such as CD10, smooth muscle actin, muscle-specific actin, S-100 protein, calponin, smooth muscle myosin heavy chain (SMMHC), and nuclear antigen p63. Currently, the combined use of more than one MEC marker is recommended to ensure accurate and reliable results [5].

## Conclusions

DOG1 seems to be similar to p63 as a myoepithelial cell marker both in normal breast tissue and in benign lesions. It is useful as a myoepithelial marker in differentiating invasive breast carcinoma and non-invasive breast lesions. There was only occasional DOG1 immunoreactivity in stromal or vascular cells, which provides an advantage compared to other cytoplasmic antigens specific to myoepithelial cells. Hence, DOG1 can be considered a good marker for differentiating benign breast lesions from malignant breast diseases.
